# Can we cure ATL: new drugs and targeting ATL stem cells

**DOI:** 10.1186/1742-4690-11-S1-O33

**Published:** 2014-01-07

**Authors:** Olivier Hermine

**Affiliations:** 1 Department of adult hematology, laboratory of physiopathology and treatment of hematological disorders, Institut Imagine, University of Sorbonne Paris Cite, Necker Hospital. Paris, France

## 

No abstract submitted

